# Cyst of the Pancreas

**Published:** 1888-04

**Authors:** D. A. K. Steele

**Affiliations:** Professor of Orthopedic Surgery in the College of Physicians and Surgeons; 1801 State Street


### CYST OF THE PANCREAS, WITH REPORT OF A CASE.

BY D. A. K. STEELE, M. D.,

Professor of Orthopedic Surgery in the College of Physicians and Surgeons.

[Read before the Chicago Medical Society.]

Senn, of Milwaukee, has so thoroughly elucidated the subject of diseases and injuries of the pancreas, that, thanks to his researches and experimental observations, the diagnosis and surgical treatment of the somewhat rare affection known as cyst of the pancreas becomes not only possible, but also safe and comparatively satisfactory.

So far, surgical literature contains reports of but seventeen cases. To this list I will add one, making eighteen on record up to date. It is a matter of satisfaction to know that the later additions to this list have all been made by American surgeons, Bull of New York, and Fenger of Chicago, preceding me in reporting cases during the past year. Case XVIII, Traumatic Cyst of Pancreas, presents the following condition and history:

Name, William Johnson; civil state, single; age, 40; occupation, police officer; nativity, Indiana, U. S.

*Family History.*—Parents, sisters, and brothers all living and in good health. No hereditary taint exists in the family.

*Previous History and Personal Habits.*—The patient had been in good general health up to the commencement of his present illness. When twelve years old he fell on his abdomen across a rail, and for the subsequent two weeks was unable to assume a recumbent position. About fifteen years ago he was struck on one side, below the ribs, during a fight. While railroading, some thirteen years ago, he was dragged on the ground and squeezed between cars, but no immediate, serious symptoms followed. His right fibula was fractured and foot dislocated outward, in 1876. He has had chancroids and bubo; but, with this exception, he gives no venereal history. He is a moderate smoker, takes an occasional drink, but never indulges in liquor to excess, and has been a police officer for the last ten years.

*History of Present Illness.*—In 1875 a swelling was noticed, occupying part of umbilical and epigastric regions. It slowly increased in size, assuming the shape of a hen's egg. It was tender on pressure, and its point of greatest prominence was located in the median line about midway between xiphoid appendix and umbilicus. At that time he was engaged as a locomotive engineer, liable to exposure and to great irregularity in the time of eating.

He does not recollect having met with any accident or fall that he could immediately associate with the appearance of the swelling, but he did suffer from "heart-burn," colicky pains and cramps at the seat of the tumor, and occasionally he also vomited some stringy mucus for a few days preceding the first sign of the swelling; his bowels remained regular. Professor Hall, of the Hahnemann Medical College, pronounced the tumor an omental cyst, and withdrew by means of the aspirator about three pints of chocolate-colored fluid, of about the consistency of porter. Some fullness of the abdomen remained, but a distinct swelling did not recur until in 1883, in August of which year Dr. Powell removed about four gallons of brownish fluid with the trochar. In the spring of 1884 the same quantity of similar fluid was removed. In November, 1885, Dr. Fenger removed with an aspirator about four and one-half gallons of porter-like fluid, some of which he applied to the integument of the patient's arm, where it produced a slight redness. In November, 1886, Dr. Powell again withdrew about the same quantity of fluid.

During all that time the patient was in good general health; his appetite was good; his digestion was normal, and his bowels regular, though his stools were perhaps a little light in color. There was no marked tenderness of the abdomen at any time, but there was considerable pain in the back, which became aggravated each time that the tumor increased in size. He felt somewhat weak for about two weeks after each removal of fluid, but after that felt well until the increasing size of the swelling produced shortness of breath and pain in the back. In 1883 he had a hydrocele on the left side of the scrotum, which was cured by iodine injections, after withdrawal of fluid. He has varicose veins of both legs, of several years' duration. There has been no swelling of the ankles at any time. His usual weight has been about 195 lbs. up to the last three months, during which he has gradually lost flesh, and now he weighs 172 lbs. The tumor is at present as large as it ever has been, and produces shortness of breath, pain in the back, pain in the left hip-joint and thigh, but no loss of appetite or noticeable change in any of the excretions; and with the exception of the loss in weight, the patient's general health seems unimpaired, and were it not for the abnormal size of his abdomen, he would present the appearance of a strong, healthy, and vigorous man.

*Physical Examination.*—Inspection reveals an enlargement of nearly the whole abdomen, its most prominent point being in the mesial line, about 5.5 inches above the umbilicus and four inches below the xiphoid appendix, when the patient is recumbent. When he is upright, the greatest prominence is a little to the right of mesial line. By percussion, there was made out a line of dullness extending from a point two inches below the left nipple, and passing four inches below the right nipple, half an inch below and parallel with the right costal arch to about two inches above the lower border of the right lumbar region, thence obliquely across the abdomen one-half inch below the umbilicus to the anterior superior spine of the left ilium. From this line and the crest of the ilium upward to a line from a point two inches below the left nipple to the lower border of the tenth rib at its origin, dullness is complete on the left side around to the spinal column. It will thus be seen that the tumor occupies the whole of the epigastric region, part of the right hypochondriac, nearly all of the right lumbar and umbilical, and the whole of the left lumbar and left hypochondriac regions. Percussion elicits dullness over the entire area. The upper border of the liver lies one-half inch below the right nipple; its left border extends two inches to the left of the right nipple; its lower border runs parallel with and an inch above the right costal arch. The space between the liver and the area of dullness gives tympanitic resonance. The apex beat is located one-half inch below the left nipple, and two inches to its right; between the area of cardiac dullness and the upper border of the tumor, resonance is slightly tympanitic. His lungs are normal. The surface of the enlargement is smooth and tense. Fluctuation is distinct over the entire area of dullness. There is limited tenderness on pressure immediately above the upper border of the tumor and in the lower part of the right lumbar region. There is no hydatid fremitus, no expansile pulsation, no auscultatory murmur, no enlargement of superficial veins of abdomen, no œdema of the lower extremities, but varicose veins of both legs exist.

October 14. *Fæces.*—Dark brown in color, of consistency of dough, medium in amount, very smooth and oily in appearance, looks as if food were well digested.

*Urine.*—Examination negative.

October 15. *Operation.*—With the assistance of Drs. N. Senn, E Powell, E. E. Babcock, and the house staff, under strict antiseptic laparotomy precautions, an incision about four inches in length and two inches to the left of the umbilicus was made through the abdominal wall, the lower angles of the incision corresponding to the site of the various aspirations previously performed. The peritoneal layers were divided on a grooved director. There were no adhesions between the peritoneum and the cyst-wall, which was very vascular and moved freely with each respiratory movement. The peritoneum was then stitched to the abdominal wall by means of silk sutures, the surfaces of the cyst-wall corresponding to the margins of the incision were lightly scratched with a blunt needle in order to excite firm cohesive inflammation, the wound packed with idoform gauze, and a heavy antiseptic dressing applied.

*The second operation* was done without any anæsthetic, Dr. E. Powell and the house staff being present. The dressing was removed and the wound found perfectly aseptic, with firm adhesions between the peritoneum and the cyst-wall, the exposed surface of which was covered with a thick grayish layer of exudation; the sutures were removed from the margins of the incision. Primary union had taken place between the incised integument and the peritoneum. In order to demonstrate with certainty that none of the abdominal viscera covered the tumor, a hypodermic needle was introduced into the supposed cyst-wall, which was found uncovered, but increased in thickness to about three-fourths of an inch, due to the abundant plastic material that had been thrown out. By means of Paquelin's cautery, at a dull red heat, a deep furrow was plowed through the middle of the surface of the cyst-wall exposed by the preliminary incision, until the wall was thin enough to permit a large trochar and cannula to be pushed through easily, without any danger of rupturing the adhesion that bound the tumor to the abdominal wall, and about 15½ pints of porter-like fluid removed, while an assistant simultaneously exercised firm pressure over the abdomen. The edges of the opening into the cyst were then seized and securely held by catch-forceps and tenacula, a piece of strong silk passed through and looped, in order to still further secure the sac from being pushed out of sight, while a double drainage-tube, about half an inch in diameter and eight inches long, one limb perforated and the other whole, was introduced. The surface of the abdomen about the incision into the tumor was anointed with iodoform vaseline, to prevent any possible digestion by the fluid, and a copious absorbent dressing applied, and firm compression secured by an elastic bandage.

The patient suffered but little pain during the operation, and felt very well at its conclusion. Was placed on liquid diet.

October 27. Soup and crackers for dinner. Dressed, looking very well. Discharge decreasing. Skin not digested. Shortened tubes half an inch. (No iodoform used, Bismuth sub nit.) Appetite increasing.

October 28. Rested better than had for several nights previous.

October 29. Dressed. Co. Glyc. powder.

October 30. Slept well.

November 1. Dressed. Skin not digested. Tubes shortened half an inch.

November 2. Slept fairly well.

November 3. Dressed.

November 5. Dressed. Tubes shortened half an inch. Cavity appears to hold about three ounces. Discharge clear, somewhat stringy, and has odor of infusion of hay.

Specimens of the fluid were submitted to Professor Gibson, of the College of Physicians and Surgeons, and to Professor Haines, of Rush Medical College, for examination.

Professor Gibson reported that "the fluid from the pancreatic cyst, is composed largely of albumen, serum, blood corpuscles, a small quantity of fatty matter indicating cholesterin, oxalate of calcium indicating oxaluric acid, sodium and potassium salts in small quantities, together with a large quantity of water. I was unable to test for digestives."

Professor Haines reported negative results.

A careful examination of the interior of the cyst with a probe and the finger shows that it involved principally the tail of the pancreas or the splenic end; also, that its interior is lined with calculous concretions, probably resulting from chemical changes in the cyst contents. At present, three and a half months after the operation, the cyst continues to discharge two or three ounces of light-colored semi-purulent fluid, with a fresh hay-like odor. The cyst, when freely distended, now has a capacity of twenty-eight ounces. The sides are thrown into irregular folds or pouches, as the result of the contraction that has taken place. The cyst-wall is very thick—half to three-quarters of an inch—and covered in places with a calculous incrustation, flakes of which can be readily removed with the finger nail. The patient's general health is good, but I am not satisfied with a cure by having established a permanent external pancreatic fistula, as advised by Senn. In this case, the sac left is too large, and the discharge too copious and annoying to the patient. I believe that we will be obliged to treat it as we would an abscess or gangrene of the pancreas, by through-and-through drainage, thereby affording perfect collapse of the sac and subsequent retraction to a small fistulous track with a minimum amount of discharge and inconvenience.

This can only be done by left-lumbar drainage, above or by the side of the kidney. The anatomical environments of the posterior wall of the cyst would preclude safe drainage in any other direction.

Senn advises, in the case of abscess, the carrying of a strong pair of curved forceps by the side of or above the kidney, and cutting down upon the point as it carries the cyst-wall and skin outwards in the loin; then to open the forceps, seize a large rubber drainage-tube and draw it into the abscess-cavity out of the anterior original incision. I do not agree with Senn in this proposition, because I believe the technique is faulty. A better plan would be to carry the drainage-tube down through the original incision to the bottom of the cyst with strong curved forceps, then to push the point against the skin until plainly felt distending it, then to make an incision down upon the cyst-wall over the end of the forceps, and lightly sear the edges of the incision with a Paquelin cautery to seal up any bleeding vessels. A strip of iodoform gauze may then be carried down to the sac-wall to act as a capillary drain for any fluid that might form a pocket in the loose connective tissue around the kidney, and finally to incise the sac over the forceps, push the *forceps and the drainage-tube through*, expand the blades of the forceps, so as to tear the incision in the sac slightly, then to close and withdraw them. In this way we run the minimum amount of risk of infection of *clean* tissue, or of separating the connection between the posterior cyst-wall and adjacent connective tissue.

The result in this case, at the date of this report, is therefore not wholly satisfactory. We have established a permanent fistula, as recommended by the highest authorities, but on account of the extreme thickness of the walls of the cyst, an ideal contraction of the cyst can not take place, and a subsequent resection of a portion of the cyst-wall around the fistulous orifice will be necessary, or else the through drainage previously suggested.

CONCLUSION.

*Remarks.*—The points of interest in this case are:

1. Its traumatic origin, being noticed a few months after a severe contusion of the body and a compression of the pancreatic region between cars.

2. Its slow growth and great size, without marked impairment of health, due probably to its originating in the tail of the pancreas; the growth extending over thirteen years (equal to the longest on record), and containing four and one-half gallons of fluid.

3. Its walls being lined with calculous incrustation, and raising the question as to whether its origin might not be due to an impacted calculus in the common duct.

4. Incision and drainage through the anterior abdominal wall not being followed by collapse and contraction of the cyst at the end of three and one-half months, raises the question of the advisability of through-and-through drainage, or excision of a part of the sac.

1801 STATE STREET.

